# Controlling Oligomer Chain Length via Ultrasonic Pretreatment in Lactic Acid Polycondensation for Enhanced Poly(lactic acid) ROP

**DOI:** 10.1021/acsomega.4c07712

**Published:** 2025-04-09

**Authors:** Nikmatuz Zahra, Endarto Yudo Wardhono, Hikmatun Ni’mah, Graecia Lugito, Tri Widjaja

**Affiliations:** † Department of Chemical Engineering, Faculty of Industrial Technology and Systems Engineering, Institut Teknologi Sepuluh Nopember, Surabaya 60111, Indonesia; ‡ Department of Chemical Engineering, Faculty of Engineering, University of Sultan Ageng Tirtayasa, Cilegon 42435, Indonesia; § Department of Chemical Engineering, Faculty of Industrial Technology, Institut Teknologi Bandung, Bandung 40132, Indonesia

## Abstract

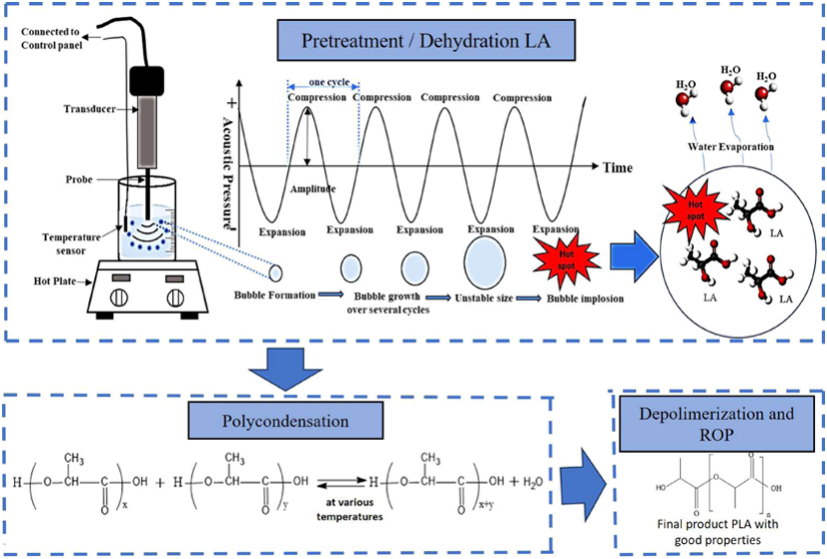

Controlling oligomer chain length in lactic acid (LA) polycondensation is crucial for producing good properties of poly(lactic acid) (PLA). This study explores the use of ultrasonic pretreatment to reduce the water content of LA, aiming to optimize the polycondensation process and enhance the quality of PLA through ring-opening polymerization (ROP). The methodology involved varying ultrasonic treatment time and power during LA pretreatment, followed by polycondensation at the optimized temperature. The study results indicate that ultrasonic pretreatment effectively reduces the water content in LA, with optimal conditions found at 90 min and 75 W, yielding the lowest water content. The polycondensation process, conducted at a gradual temperature of 150 °C followed by 180 °C, resulted in the highest yield of 92.75% and a molecular weight of 25,126 g/mol for the oligomers. Ultrasonic pretreatment enhances water removal efficiency, reduces byproduct formation, and increases oligomer reactivity, resulting in higher-purity oligomers and improved chain length control. During the ROP stage, oligomers prepared through ultrasonic pretreatment produced PLA with a higher molecular weight and crystallinity.

## 1 Introduction

The increasing use of conventional plastics, either petroleum-based or nonbiodegradable, or both, has led to severe environmental pollution.^1,2^ Poly(lactic acid) (PLA) has emerged as a sustainable alternative to petroleum-based plastics.^3,4^ Unlike nonbiodegradable plastics, PLA offers two significant advantages: it is derived from renewable resources and can be composted at the end of its life cycle.^5,6^ As a biodegradable polymer, PLA can be broken down by soil bacteria, potentially mitigating environmental pollution.^7^

PLA is a linear aliphatic thermoplastic polyester made from lactic acid (LA) monomer that has good mechanical properties, thermal stability, processability, and a low environmental impact.^8−10^ PLA is widely applied in the food packaging, textile, agricultural, and medical industries.^5^ It is safe for all food packaging applications and has been classified as generally regarded as safe (GRAS) by the US Food and Drug Administration (FDA).^11^ Several main routes exist for synthesizing PLA, including direct polycondensation (DP), ring-opening polymerization (ROP), and azeotropic polymerization.^12^ Among those methods, ROP is considered the most popular and practical way to create high-molecular-weight PLA with narrow molecular weight distribution.^5,13^ Stannous octoate (Sn(Oct)_2_) was discovered to be the optimum catalyst when using the ROP approach.^14,15^

ROP of lactide involves four stages: pretreatment of lactic acid (LA), polycondensation of LA, depolymerization of oligomers, and the ROP process itself.^16,17^ In the first stage, LA is desiccated to lower the water content, which is essential for subsequent reactions. This is followed by the polycondensation of LA, resulting in low-molecular-weight oligomers or prepolymers. These oligomers undergo catalytic depolymerization to form lactides through internal transesterification in a backbiting reaction. Finally, the lactide ring is opened with the aid of a catalyst, yielding high-molecular-weight PLA.^13^ The chain length of the oligomers plays a significant role in determining the properties of PLA, making its control during the polycondensation stage crucial for achieving the desired characteristics.

Research on the synthesis of lactic acid oligomers has historically focused on optimizing polycondensation conditions such as temperature, pressure, and the use of catalysts.^13^ The chain length of oligomers and their hydrophobicity influence the enhancement of bioplastic properties.^18^ Previous studies have employed traditional polycondensation methods. For example, Rahmayetty et al. carried out polycondensation at various temperatures without catalysts and with conventional heating pretreatment,^19^ while Triwulandari et al. varied the polycondensation time of lactic acid without catalysts.^18^ These processes often require long times and high temperatures; the yields obtained are not very high yet, and the purity of the oligomers is poor, making it difficult to control the chain length of the oligomers. A major gap in the literature is the difficulty in precisely controlling the chain length of oligomers using conventional polycondensation methods. Residual catalysts complicate achieving specific chain lengths and the desired properties of PLA. Additionally, traditional methods often overlook the potential of alternative energy sources, such as ultrasonic energy, to enhance the synthesis process.

Ultrasonic wave irradiation is an efficient process for synthesizing organic products due to its numerous advantages, including reduced time and cost, milder reaction conditions, and higher yields.^20^ Ultrasound induces cavitation, involving the formation, growth, and collapse of bubbles in a liquid.^21^ This process generates intense local heating and high shear forces,^22^ significantly enhancing water removal efficiency. In this study, ultrasound was employed in the LA pretreatment process. The lactic acid obtained from this process was polycondensed at various temperatures to achieve the highest oligomer yield. These oligomers were then used in the subsequent step of synthesizing PLA via ROP.

Temperature is another crucial parameter in the polycondensation. The reaction temperature affects both the rate of polymerization and the molecular weight of the resulting oligomers.^23^ By systematically varying the temperature, better control over oligomer chain length can be achieved. Higher temperatures may accelerate the condensation process but risk forming unwanted byproducts or degrading the oligomers.^13,24^ This study aims to investigate the combined effects of ultrasonic pretreatment and temperature optimization to refine the control over oligomer chain length, thereby enhancing the subsequent ROP process for PLA production.

This research offers a novel approach to synthesizing lactic acid oligomers with precise control over their chain length, which is critical for producing PLA with good properties such as optimal molecular weight and desired crystallinity. Integrating ultrasonic pretreatment with temperature optimization in the polycondensation step presents a promising advancement in PLA production, potentially leading to more efficient and cost-effective processes with reduced environmental impact.

## 2 Experimental Section

### 2.1 Materials

Lactic acid (LA) (88–92%), chloroform (CHCl_3_) (molar mass: 119.38 g/mol, ρ = 1.49 g/cm^3^ at 25 °C), and tin(II) chloride dihydrate (SnCl_2_·2H_2_O) (*M̅*_w_: 225.63 g/mol, ρ = 2.71 g/cm^3^ at 20 °C) were all acquired from Merck, Darmstadt, Germany. Stannous octoate (Sn(Oct)_2_) (92.5–100%) and methanol (CH_3_OH) (purity: 98%) were purchased from Sigma-Aldrich, Singapore. Nitrogen was supplied from the Industrial Gas Samator, Mojokerto, Indonesia. These components were not further purified before usage.

### 2.2 Research Methodology

The research methodology consists of several stages. LA first undergoes pretreatment, followed by polycondensation to form oligomers. These oligomers are then depolymerized into lactide, which is finally polymerized through ring-opening polymerization (ROP) to produce PLA.

#### 2.2.1 Ultrasonic Pretreatment

Lactic acid (50 mL) was heated to 100 °C in a glass beaker. An ultrasonic cell distributor probe (UCD-P01, Biobase, Shandong, China) operating at 20–30 kHz with a 5 mm probe size was immersed in the lactic acid. Pretreatment time was varied from 30 to 120 min, and ultrasonic power from 50 to 150 W. The density of the dehydrated lactic acid was measured, and water content was analyzed using a Karl Fischer instrument.

#### 2.2.2 Polycondensation of LA

The pretreated LA was placed in a four-neck flask equipped with a magnetic stirrer, thermometer, and condenser. Polycondensation was carried out to form oligomers at various constant temperatures of 150, 160, 170, and 180 °C, each for a duration of 4 h. A separate trial was conducted where the temperature was maintained at 150 °C for 2 h, followed by an increase to 180 °C for another 2 h, resulting in a total of five trials at the temperatures of 150, 160, 170, 180, and the 150–180 °C range. A nitrogen gas stream was used to drive off air vapor, and vacuum pressure was applied while stirring at 150 rpm. The yield of the formed oligomers was calculated. Functional groups and molecular structure were determined using Fourier transform infrared spectroscopy (FTIR) and H NMR. Molecular weight was also calculated using the Mark–Houwink–Sakurada equation with a viscosity approach.

#### 2.2.3 Depolymerization of Oligomers

The oligomers produced in the polycondensation process were heated at 210 °C under vacuum and stirred at 150 rpm with 0.2% (w/w) SnCl_2_·2H_2_O catalyst. The oligomers were heated until no distillate was produced. The vacuum valve was slowly closed after the process was completed. The lactide was then collected in a sample container. The lactide obtained was then characterized using FTIR and H NMR.

#### 2.2.4 Ring-Opening Polymerization of Lactides

The lactide formed during the depolymerization process was mixed with 0.2% (w/w) stannous octoate (Sn(Oct)_2_) catalyst and heated at 140 °C for 4 h. The PLA was then dissolved in chloroform and precipitated with methanol before being filtered and dried. The dried PLA was then tested for molecular weight using the viscosity approach, functional groups and molecular structures using FTIR and H NMR, and crystallinity using X-ray diffraction (XRD).

#### 2.2.5 Characterization

The Karl Fisher instrument (Karl Fischer Moisture Titrator MKV-710S, Kyoto Electronics Manufacturing Co., Ltd., Kyoto, Japan) was used to measure the moisture content in melted LA. A Fourier transform infrared spectrometer (FTIR, Thermo Scientific Nicolet iS10, Madison, WI) was used to analyze the functional groups of oligomers, lactides, and PLA. Proton nuclear magnetic resonance (H NMR JEOL ECS-400 spectrometer operating at 400 MHz, Tokyo, Japan) was used to determine the molecular structure of oligomers, lactides, and PLA. Meanwhile, X-ray diffraction (XRD X’Pert PRO PANalytical, Almelo, Netherlands) was used to determine the degree of crystallinity of the resulting PLA products. The crystallinity index was calculated according to eq 1
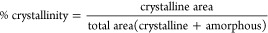
1

Viscosity is affected by the solutes in the liquid. The addition of polymers can increase the viscosity of the liquid.^25^ Intrinsic viscosity [η] is an analog of the virial coefficient (and has a dimension of 1/concentration).^26^ Molecular weight was analyzed by measuring the flow times of ethyl acetate (*t*_0_) and PLA in ethyl acetate (*t*) at concentrations of 0.2, 0.3, and 0.4% using an Oswald viscometer. Intrinsic viscosity was obtained by plotting PLA concentration versus reduced viscosity and extrapolating to zero concentration. The molecular weight was then calculated using the Mark–Houwink–Sakurada equation.^27^

## 3 Results and Discussion

### 3.1 Effect of Ultrasonic Pretreatment

The LA pretreatment attempts to remove the water present in LA. During the polycondensation, water and LA are in equilibrium in the oligomer formation reaction. Consequently, if the water is not removed, the formation of oligomers is restricted.^28^ Water is unfavorable in this polymerization step because it promotes chain transfer, resulting in low-molecular-weight PLA. Removing water from LA is an important step that can influence the characteristics of the final PLA product obtained.^29^ This study employed ultrasonic waves for LA pretreatment, varying both time and power.

**Figure 1:**
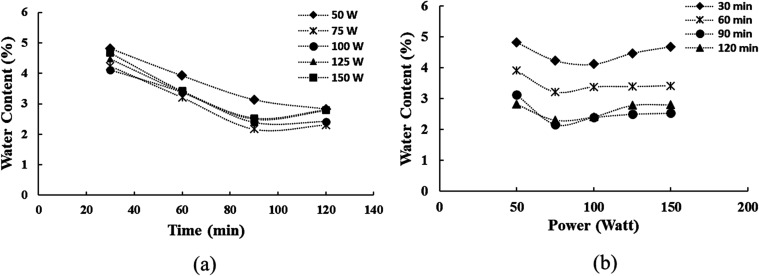
(a) Graph of water content vs ultrasonic time at various power levels. (b) Graph of water content vs ultrasonic power at various times.

Figure 1 demonstrates that water content decreases with increasing ultrasonic time, but it rises slightly at 120 min, possibly due to water reabsorption or LA degradation. The optimal condition was found to be 90 min at 75 W, resulting in the lowest water content. Higher power levels increased water content, likely due to localized overheating, which can lead to thermal degradation of LA.^30^

The optimal pretreatment conditions were also compared with the conventional pretreatment process without ultrasonics under vacuum conditions. The results of the water content and density comparison are presented in Table 1.

**Table 1:** Density and Water Content of LA

LA	density (g/mL)	water content (%)
before pretreatment	1.189 ± 0.0003	14.6 ± 0.17
after conventional pretreatment	1.205 ± 0.005	6.3 ± 0.18
after ultrasonic pretreatment	1.223 ± 0.002	2.73 ± 0.1

Table 1 indicates a reduction in LA density following the water removal process, with ultrasonic pretreatment resulting in a density close to the literature value of 1.224 g/mL for pure LA.^28^ This increase in density indicates a higher concentration of LA relative to water, post-removal. Karl Fischer analysis confirmed that water content decreased with increasing density in LA, with ultrasonic pretreatment achieving lower water content than vacuum or conventional methods. Thus, ultrasonic pretreatment proves more effective than vacuum pretreatment for the removal of water from LA.

**Scheme 1:**
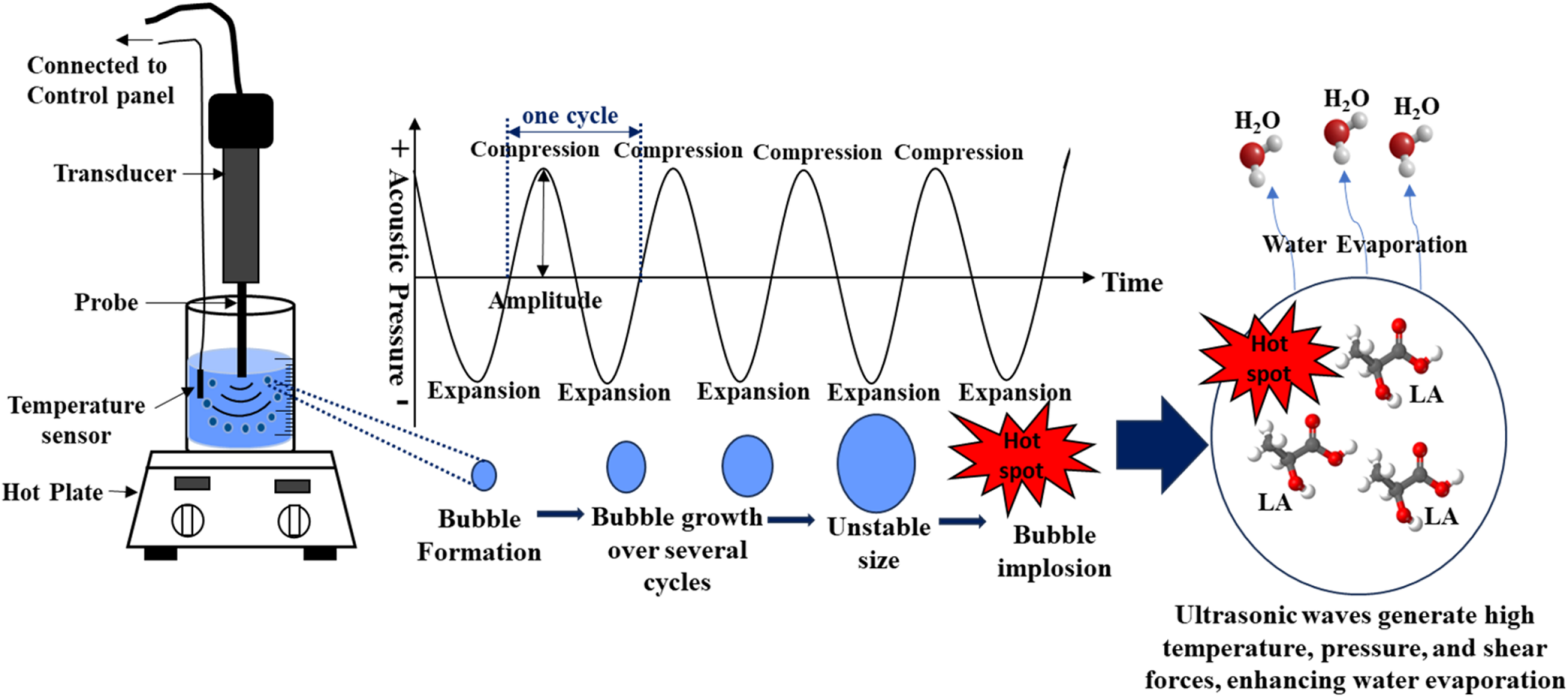
Illustration of the LA Pretreatment Mechanism Assisted by Ultrasonic Waves

The ultrasonic pretreatment mechanism of LA follows the cavitation theory illustrated in Scheme 1. Ultrasonic cavitation initiates in LA with the formation of small bubbles induced by negative pressure from ultrasonic waves.^21^ These bubbles progress through several phases: nucleation, where small bubbles initially form due to the low-pressure conditions created by ultrasonic waves; growth, as these bubbles expand and increase in size during each cycle of ultrasonic wave action; an unstable size phase, where bubbles reach a critical size and exhibit fluctuations in size and shape; and finally, a collapse phase, where bubbles implode suddenly under high-pressure conditions,^31,32^ generating localized hot spots characterized by extremely high temperature and pressure.^33^ This rapid collapse generates intense shear forces within the liquid medium, which are crucial for breaking down water molecules and accelerating the evaporation process. The high-energy zones and shear forces created during cavitation enhance mass transfer,^34^ facilitating more efficient movement of water molecules toward the surface for evaporation.^30^

The significant reduction in water content observed with optimal ultrasonic pretreatment directly supports the goal of controlling oligomer chain length. Lower moisture content in melted LA minimizes unwanted chain transfer reactions during polycondensation,^29^ forming longer and more uniform oligomer chains. This control over oligomer chain length is crucial for producing high-quality PLA with desirable properties.^35^ As a conclusion, ultrasonic pretreatment plays a pivotal role in achieving the targeted molecular structure and properties of the final PLA product.

### 3.2 Effect of Polycondensation Temperatures

Polycondensation occurs as two lactic acid monomers combine to produce dimers, trimers, and oligomers, generating water vapor by incorporating two carboxylic acid groups into an ester group.^36^ At this stage, lactic acid obtained from the optimal ultrasonic pretreatment conditions is polycondensed with various temperature variations (150–180 °C) and a processing time of 4 h under vacuum conditions and with nitrogen gas flowing to encourage water vapor removal as a byproduct of the polycondensation reaction. At this stage, a solid catalyst is not employed since the acidic feed can function as a catalyst in the polycondensation reaction.

The primary focus of this investigation was removing feedwater to steer the polycondensation reaction toward oligomer formation. Table 2 presents the yield of oligomers obtained at different temperatures during the polycondensation process, along with the molecular weight determined using the viscosity approach.

**Table 2:** Oligomer Yields from Polycondensation Process with Various Temperatures, Following Ultrasonic Pretreatment

polycondensation temperature (°C)	oligomer yield (%)	*M̅*_w_ viscosity approach (g/mol)	lactide byproduct
150	84.99 ± 0.01	13,952	not formed
160	87.25 ± 0.02	15,728	formed
170	87.66 ± 0.01	16,744	formed
180	86.54 ± 0.002	21,468	formed
sequential temperature (150 °C then 180 °C)	92.75 ± 0.03	25,126	formed

**Scheme 2:**
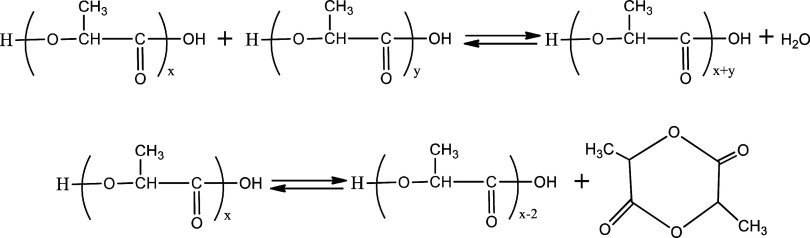
Two Reactions Involved in the Polycondensation Process of LA

Table 2 shows the percent yield and molecular weight of oligomers increased with increasing temperature, but there was a slight decrease at 180 °C. The highest yield and molecular weight of oligomers was obtained from the polycondensation at sequential temperatures of 150 °C for 2 h, followed by 180 °C for 2 h, with a yield of 92.75% and a molecular weight of 25,126 g/mol. Lactide byproducts are formed at polycondensation temperatures above 160 °C and during polycondensation with a gradual temperature increase, but not at 150 °C. Lactide byproducts form as a result of interactions between lactic acid molecules. Groot et al. suggest that lactic acid molecules, which feature hydroxyl (−OH) and carboxylic acid (−COOH) functional groups, undergo esterification reactions influenced by both intermolecular and intramolecular interactions. During polycondensation, intermolecular interactions among lactic acid molecules primarily lead to the formation of dimers, which can further react to produce trimers and higher oligomers, accompanied by the release of water molecules (H_2_O). This reaction involves the formation of an ester bond between the carboxylic acid group of one lactic acid molecule and the hydroxyl group of another, resulting in the production of dimers and, as the reaction progresses, additional oligomers. In contrast, intramolecular interactions convert lactic acid dimers into lactides.^37^ These two reactions are illustrated in Scheme 2.

**Figure 2:**
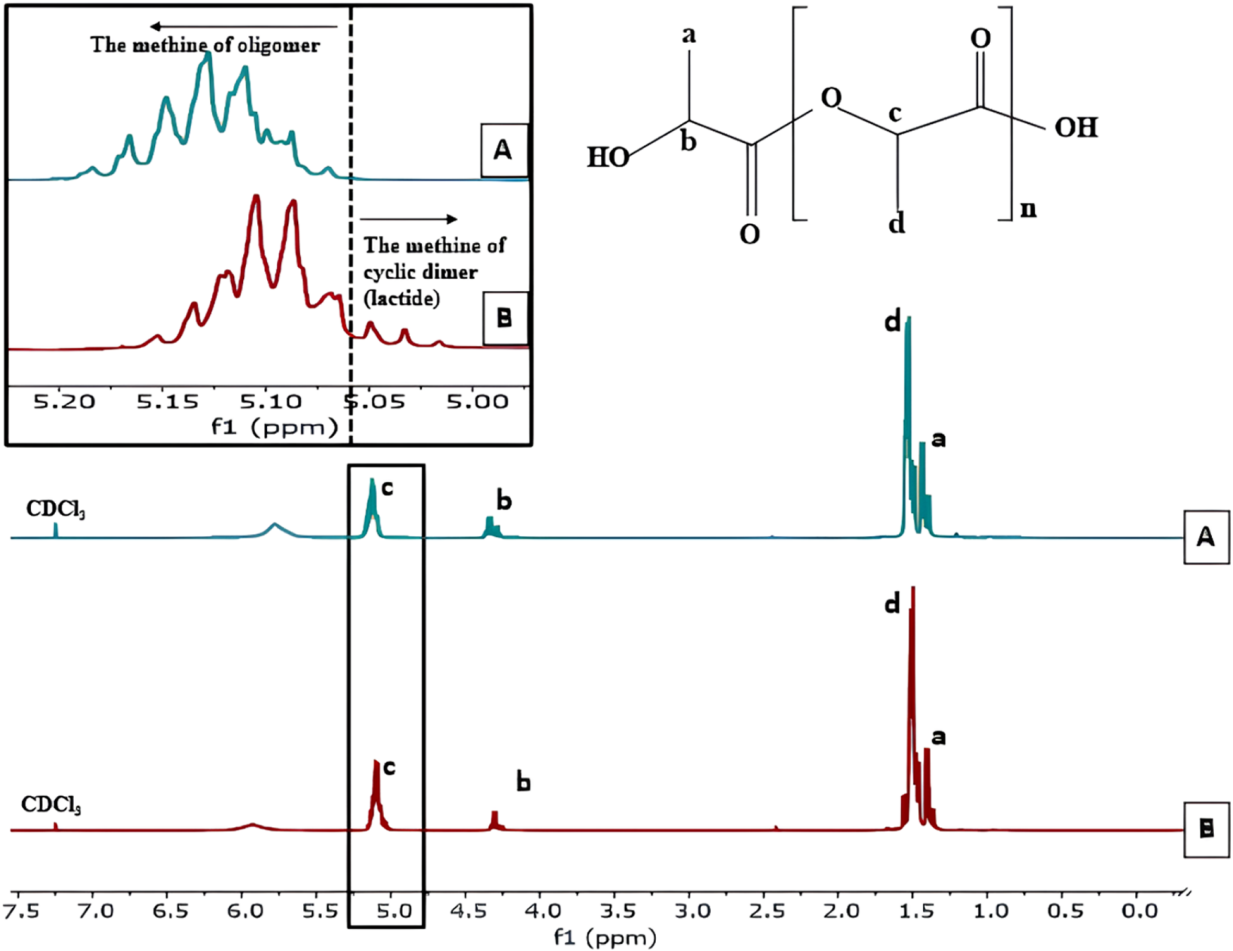
H NMR spectra of oligomer at gradual temperature (A) with ultrasonic pretreatment (B) without ultrasonic.

The H NMR (proton nuclear magnetic resonance) spectra of the oligomer at optimal temperature conditions, obtained from both processes with ultrasonic pretreatment and without ultrasonic pretreatment, are shown in Figure 2A,B. Both spectra exhibit the same singlet signal at a chemical shift value of 7.3 ppm, indicating the use of CDCl_3_ as the solvent.^38^ Additionally, a quartet signal is observed at a chemical shift between 5.06 and 5.2 ppm in both H NMR spectra of the lactic acid oligomer, whether subjected to ultrasonic pretreatment or not. This quartet signal signifies the presence of hydrogen atoms at the carboxyl end group and the hydrogen from the methine group (−CH–CH_3_).^18^ The H NMR spectrum of the lactic acid oligomer with ultrasonic pretreatment shows a doublet signal at a chemical shift range of 1.47–1.59 ppm, while the spectrum for PLA without ultrasonic pretreatment shows a doublet signal at a chemical shift range of 1.42–1.52 ppm. This doublet signal indicates the presence of hydrogen in the methyl group (−CH_3_).^39^ Moreover, at a chemical shift of 5.06–5.00 ppm, a small intensity signal indicates the presence of lactide methine proton.^18^ Other chemical shifts at 4.2–4.37 ppm indicate methine protons connected to the hydroxyl end group’s protons, and at 1.33–1.41 ppm, they indicate methyl protons of the lactic acid oligomer adjacent to the hydroxyl group.^39^ Overall, the H NMR spectra show consistent signals between samples with and without ultrasonic pretreatment, with minor variations in chemical shifts providing detailed information about the structure of the lactic acid oligomer and the interactions between atoms within the molecule.

The H NMR analysis is performed by integrating the peaks in the spectrum to determine the percentage of oligomers and the lactide byproduct. The chemical shift in the range of 5.0–5.06 ppm indicates the methine proton of lactide, while the chemical shift in the range of 5.06–5.22 ppm indicates the methine proton of the oligomer, as shown in Figure 2.

Additionally, the degree of polymerization (DP) and number-average molecular weight (*M̅_n_*) are determined based on the integration of the proton signals from the polymer end groups compared to the integration of the proton signals from the polymer repeating units.^40^*M̅_n_* can be calculated using eq 2
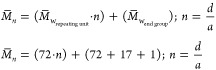
2

**Figure 3:**
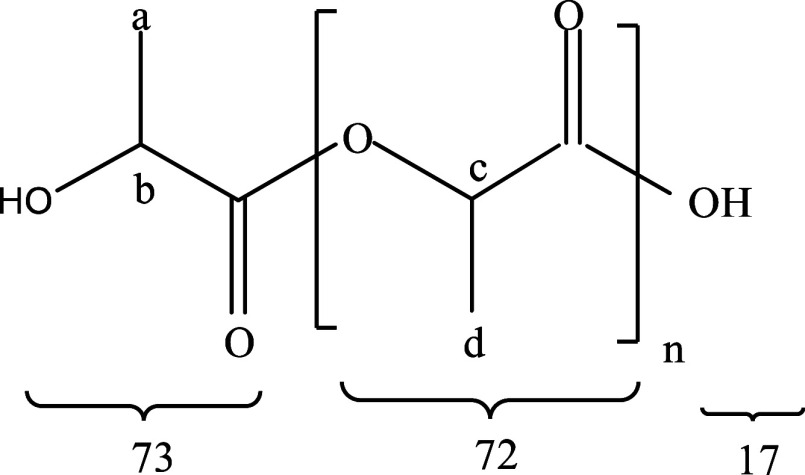
Structure of lactic acid oligomers and determination of average molecular weight using H NMR final group analysis.

In eq 2, *n* represents the number of repeating units and is determined by the ratio of the integration values of the NMR peaks for moiety “*d*” (the repeating units) and moiety “*a*” (the end groups), as shown in Figure 3.

The percentages of lactide and oligomer, as well as the *M̅_n_* and DP results based on H NMR analysis, are presented in Table 3.

**Table 3:** Percentages of Lactic Acid Oligomer, Lactide, Results of *M̅*_*n*_, and DP Based on H NMR Analysis

sample lactic acid oligomer	% oligomer	% lactide	DP	*M̅_n_*
with ultrasonic pretreatment	99.8	0.2	8.33	689.76
with conventional pretreatment	72.53	27.47	4.9	442.8

Table 3 shows that ultrasonic pretreatment of lactic acid results in higher oligomer purity compared to conventional pretreatment without ultrasonics and produces a significantly lower amount of byproduct lactide, at only 0.2%. This improved purity results from the effectiveness of ultrasonics in reducing water content, which in turn minimizes chain transfer reactions and promotes a higher rate of polycondensation. Ultrasonic cavitation creates high-energy zones and shear forces that accelerate water evaporation,^30^ facilitating a more efficient reaction and leading to oligomers with longer chain lengths. The degree of polymerization (DP) is indicative of the polymer chain length, where a higher DP correlates with longer oligomer chains.^41^ As a result, reduced water content and increased reaction efficiency contribute to higher DP and average molecular weight (*M̅*_*n*_) of the oligomers, ultimately reflecting improved oligomer purity.

### 3.3 Products Characterization

**Figure 4:**
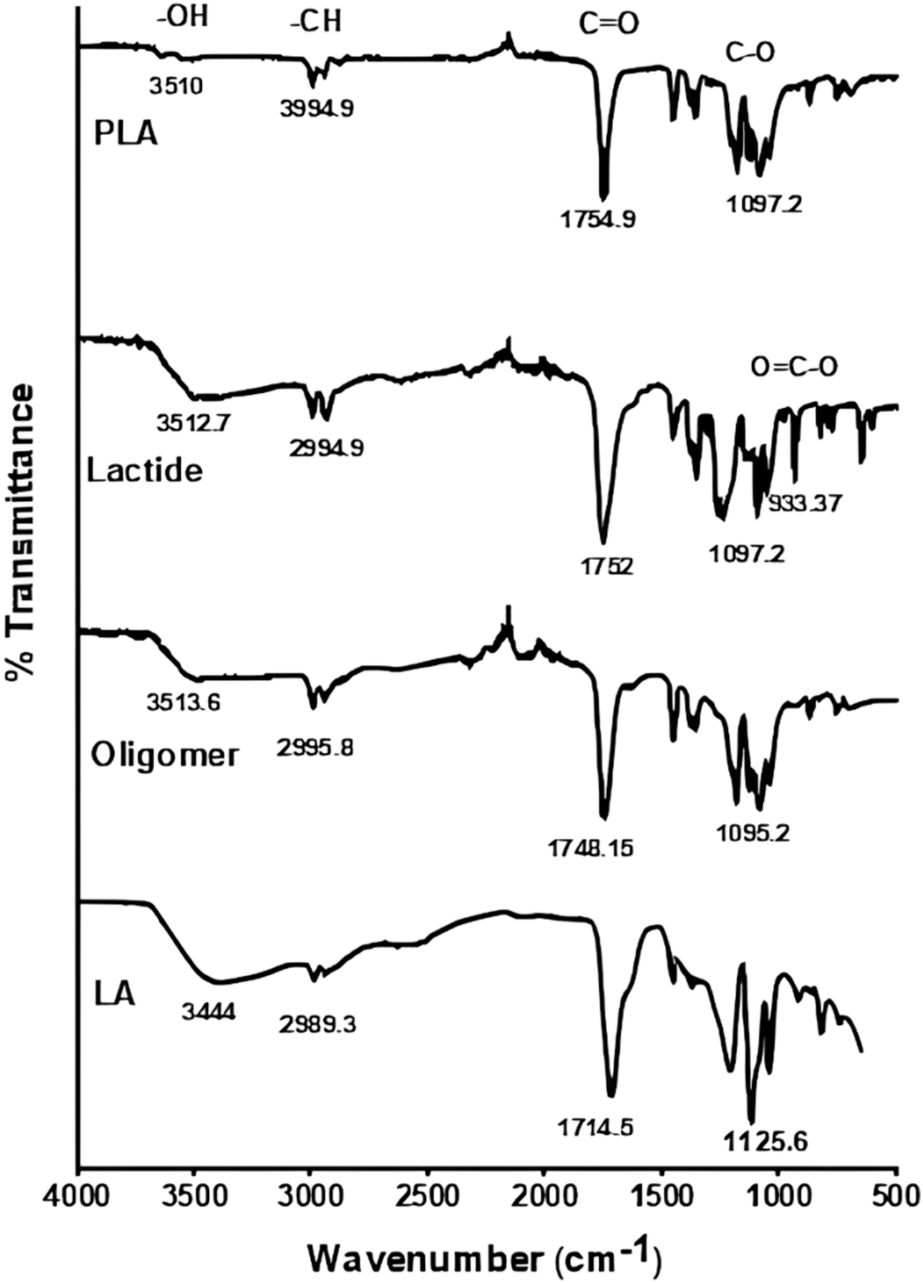
FTIR spectra of lactic acid, oligomer at gradual temperature, lactide, and PLA.

**Figure 5:**
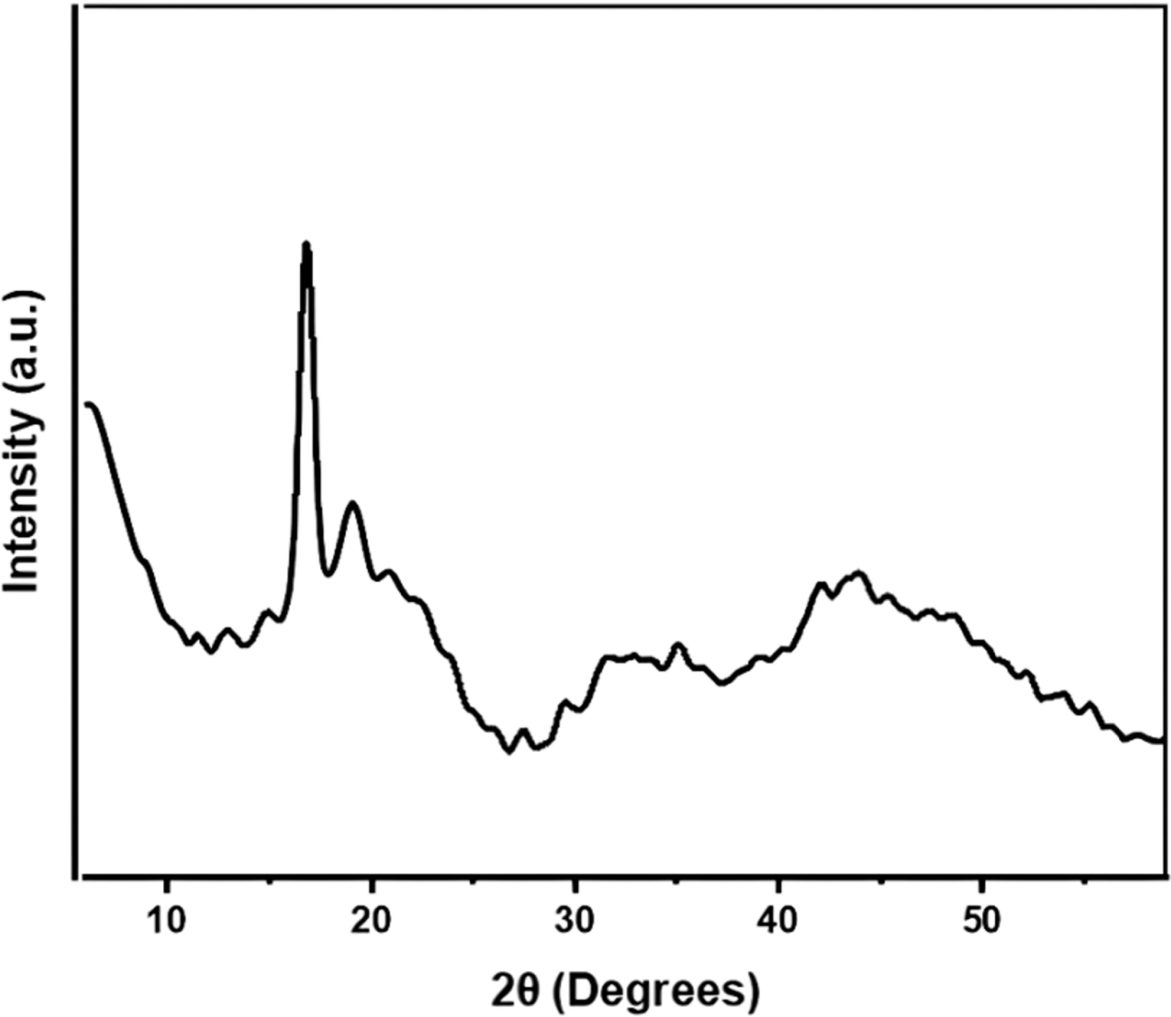
XRD diffractogram of PLA.

**Figure 6:**
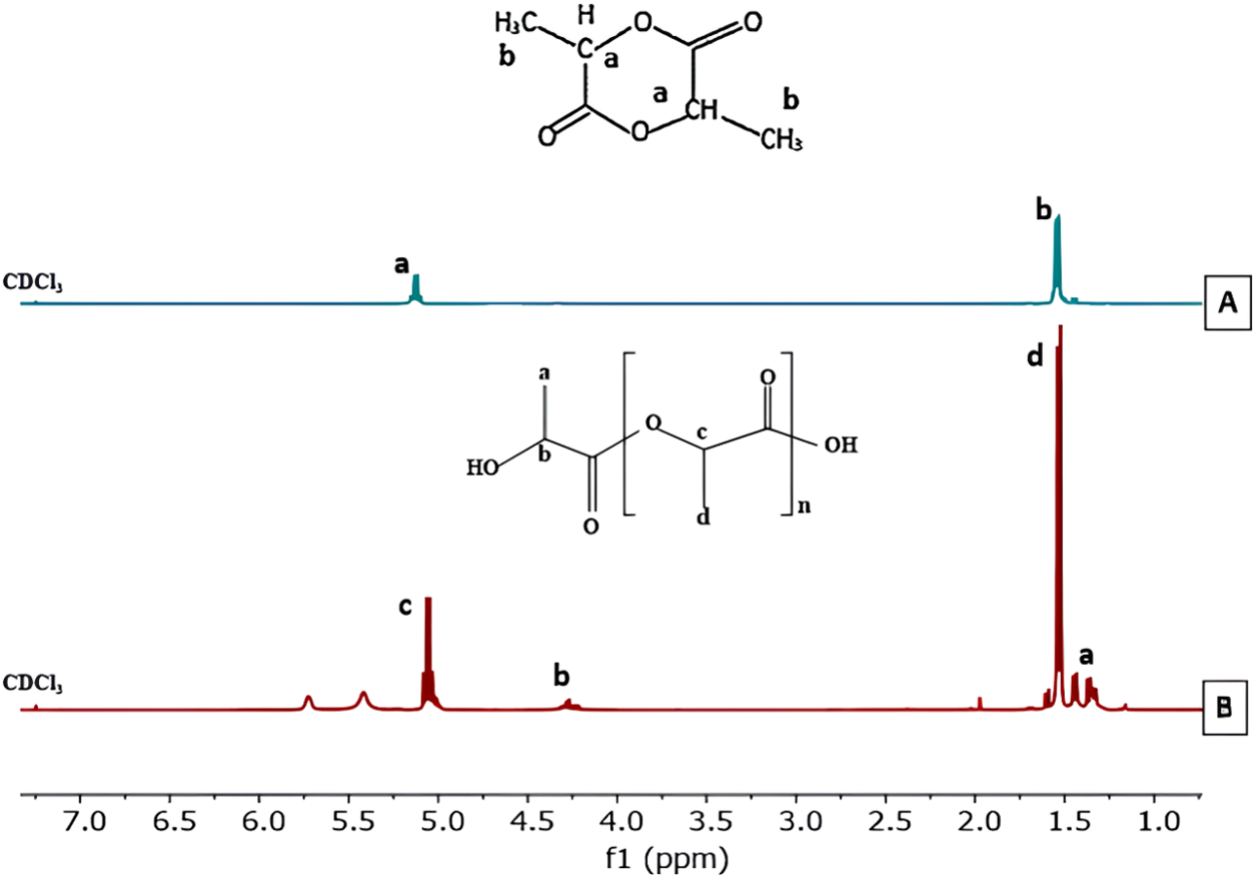
H NMR spectra of (A) lactate and (B) PLA.

Oligomers synthesized under optimal temperature conditions undergo depolymerization and polymerization via ROP. The resulting PLA was then characterized qualitatively using FTIR and H NMR. Additionally, quantitative analysis was carried out using XRD, and viscosity approach for molecular weight, as illustrated in Figures 4–6 and Table 4. These analysis techniques provide a comprehensive understanding of the structural and quantitative aspects of the obtained PLA.

**Table 4:** Molecular Weight Result of PLA

sample PLA	*M̅*_w_ (g/mol)
with ultrasonic pretreatment	67,228
with conventional pretreatment	41,076

Fourier transform infrared spectroscopy (FTIR) is a widely used technique to identify functional groups in materials using an infrared beam. Figure 4 shows the FTIR spectra for LA, oligomer at gradual temperature, lactide, and PLA, where all of these compounds have the same functional group; there is no significant change in wavenumber. The hydroxyl group −OH is shown at about 3200–3600 cm^–1^, while the carbonyl C=O is at about 1735–1750 cm^–1^. The peak of the carbonyl group shifts when lactic acid is transformed into oligomers, and the −OH stretching vibration in the oligomer noticeably decreases. The drop in −OH absorbance is due to the ultrasonic pretreatment and polycondensation processes, which use the OH groups to create the ester bond with the acid group. Peaks appeared at wavenumbers 933.3781 cm^–1^ in lactide that did not appear in oligomers previously. The appearance of wavenumber peaks indicates the formation of O=C–O lactide rings.

Figure 5 shows the XRD diffractogram results, which show two 2θ diffraction peaks at around 16.8 and 19.12°, which are consistent with previous studies.^42,43^ The crystallinity was calculated quantitatively using XRD data. The width of the diffraction peaks reveals information about the crystal structure of PLA, with a wider peak indicating more crystallized PLA.^28^ Moreover, eq 1 shows that the result of % crystallinity of the PLA products produced in this study is 42.5%. This calculation was performed using the Gaussian method to analyze the diffraction peaks in the XRD pattern and determine the crystalline proportion in the sample. This value is in line with theoretical expectations, confirming that PLA is a semicrystalline polymer.^44^ Crystallinity is affected by various factors, including molecular weight, temperature, and processing history.^45^ A high percentage of crystallinity indicates a high molecular weight of the polymer produced.

The H NMR spectra for lactide and PLA, as shown in Figure 6, reveal distinct chemical shifts. For lactide, the H-doublet signals appear at 1.52–1.54 ppm, while the H-quartet signals appear at 5.03–5.06 ppm. In contrast, for PLA, the H-doublet signals are found at 1.44–1.46 and 1.53–1.55 ppm, and the H-quartet signals are observed at 5.10–5.14 and 4.32–4.34 ppm. The H-doublet signals indicate methyl groups, while the H-quartet signals indicate methine groups in both compounds. These results confirm that the polycondensation, depolymerization, and ROP processes were successfully carried out, aligning with previous studies.^28,46,47^

Table 4 shows the molecular weight of PLA resulting from the depolymerization and polymerization of oligomers under optimal temperature conditions, using two types of pretreatments which are ultrasonic and conventional. The molecular weight (*M̅_w_*) values obtained were 67,228 g/mol for the ultrasonic pretreatment and 41,076 g/mol for the conventional pretreatment. These results indicate that ultrasonic pretreatment followed by polycondensation at optimal temperatures can produce PLA with a higher molecular weight. This is because ultrasonic pretreatment is capable of producing oligomers with a higher degree of polymerization and greater purity. This value is consistent with the literature by Hu et al., which states that the molecular weight of PLA produced through the ROP method starts from 2 × 10^4^ g/mol.^13^

## 4 Conclusions

This study demonstrates the effectiveness of ultrasonic pretreatment in enhancing lactic acid (LA) polycondensation for poly(lactic acid) (PLA) production via ring-opening polymerization (ROP). Ultrasonic pretreatment significantly reduced water content and minimized chain transfer reactions, enabling the formation of longer oligomer chains. Under optimal conditions (90 min at 75 W), the process yielded high-purity oligomers with minimal lactide byproducts. Polycondensation with a gradual temperature increase from 150 to 180 °C produced PLA with superior molecular weight and crystallinity compared to conventional methods. This approach offers a cost-effective and sustainable strategy for synthesizing PLA with desirable properties, contributing to advancements in bioplastic production.

## References

[ref1] Judawisastra H.; Sitohang R. D. R.; Taufiq D. I.; Mardiyati M. The Fabrication of Yam Bean (Pachyrizous Erosus) Starch Based Bioplastics. Int. J. Technol. 2018, 9, 345–352. 10.14716/ijtech.v9i2.1129.

[ref2] Nizamuddin S.; Jabbar A.; Chen C.; Arif M.; Mujawar N. Bio-Based Plastics, Biodegradable Plastics, and Compostable Plastics: Biodegradation Mechanism, Biodegradability Standards and Environmental Stratagem. Int. Biodeterior. Biodegrad. 2024, 195 (April), 105887 10.1016/j.ibiod.2024.105887.

[ref3] Surip S. N.; Jaafar W. N. R. W. Comparison Study of The Bio-Degradation Property of Polylactic Acid (PLA) Green Composites Reinforced by Kenaf Fibers. Int. J. Technol. 2018, 9, 1205–1215. 10.14716/ijtech.v9i6.2357.

[ref4] Sachdeva G.; Bamal Y.; Ladan A.; Tiwari O. S.; Rawat V.; Yadav P.; Verma V. P. Calixarene-Metal Complexes in Lactide Polymerization: The Story so Far. ACS Omega 2023, 8, 13479–13491. 10.1021/acsomega.2c08028. 37091416 PMC10116533

[ref5] Teixeira S.; Eblagon K. M.; Miranda F.; Pereira M. F. R.; Figueiredo J. L. Towards Controlled Degradation of Poly(Lactic) Acid in Technical Applications. J. Carbon Res. 2021, 7 (2), 42 10.3390/c7020042.

[ref6] Qi Y.; Ma H.-L.; Du Z.-H.; Yang B.; Wu J.; Wang R.; Zhang X.-Q. Hydrophilic and Antibacterial Modification of Poly(Lactic Acid) Films by Γ-ray Irradiation. ACS Omega 2019, 4, 21439–21445. 10.1021/acsomega.9b03132. 31867539 PMC6921625

[ref7] Satriyatama A.; Rochman V. A. A.; Adhi R. E. Study of the Effect of Glycerol Plasticizer on the Properties of PLA/Wheat Bran Polymer Blends. IOP Conf. Ser. Mater. Sci. Eng. 2021, 1143, 012020 10.1088/1757-899X/1143/1/012020.

[ref8] Zbigniew O.; Jalbrzykowski M.; Mystkowska J.; Romanczuk E.; Osiecki T. Mechanical and Thermal Properties of Polylactide (PLA) Composites Modified with Mg, Fe, and Polyethylene (PE) Additives. Polymers 2020, 12 (12), 2939 10.3390/polym12122939. 33316956 PMC7763237

[ref9] Lololau A.; Soemardi T. P.; Purnama H.; Polit O. Composite Multiaxial Mechanics: Laminate Design Optimization of Taper-Less Wind Turbine Blades with Ramie Fiber-Reinforced Polylactic Acid. Int. J. Technol. 2021, 12 (6), 1273–1287. 10.14716/ijtech.v12i6.5199.

[ref10] Trivedi A. K.; Gupta M. K.; Singh H. Advanced Industrial and Engineering Polymer Research PLA Based Biocomposites for Sustainable Products: A Review. Adv. Ind. Eng. Polym. Res. 2023, 6 (4), 382–395. 10.1016/j.aiepr.2023.02.002.

[ref11] Jamshidian M.; Tehrany E. A.; Imran M.; Jacquot M.; Desobry S. Poly-Lactic Acid : Production, Applications, Nanocomposites, and Release Studies. Compr. Rev. Food Sci. Food Saf. 2010, 9, 551–571. 10.1111/j.1541-4337.2010.00126.x. 33467829

[ref12] de França J. O. C.; da Silva Valadares D.; Paiva M. F.; Dias S. C. L.; Dias J. A. Polymers Based on PLA from Synthesis Using D,L-Lactic Acid (or Racemic Lactide) and Some Biomedical Applications: A Short Review. Polymers 2022, 14 (12), 2317 10.3390/polym14122317. 35745893 PMC9229942

[ref13] Hu Y.; Daoud W. A.; Cheuk K. K. L.; Lin C. S. K. Newly Developed Techniques on Polycondensation, Ring-Opening Polymerization and Polymer Modification: Focus on Poly(Lactic Acid). Materials 2016, 9 (3), 133 10.3390/ma9030133. 28773260 PMC5456738

[ref14] Ibrahim N.; Shamsuddin A. N. High Molecular Weight of Polylactic Acid (PLA):A Review on the Effect of Initiator. Malays. J. Chem. Eng. Technol. 2021, 4 (1), 15–23. 10.24191/mjcet.v4i1.12906.

[ref15] Li F.; Rastogi S.; Romano D. Synthesis of Ultrahigh Molecular Weight PLAs Using a Phenoxy-Imine Al(III) Complex. ACS Omega 2020, 5, 24230–24238. 10.1021/acsomega.0c01952. 33015439 PMC7528193

[ref16] de Albuquerque T. L.; Júnior J. E. M.; de Queiroz L. P.; Ricardo A. D. S.; Rocha M. V. P. Polylactic Acid Production from Biotechnological Routes: A Review. Int. J. Biol. Macromol. 2021, 186 (July), 933–951. 10.1016/j.ijbiomac.2021.07.074. 34273343

[ref17] Horváth T.; Marossy K.; Szabó T. J. Ring - Opening Polymerization and Plasticization of Poly (L-Lactic) Acid by Adding of Glycerol - Dioleate. J. Therm. Anal. Calorim. 2021, 147 (3), 2221–2227. 10.1007/s10973-020-10540-1.

[ref18] Triwulandari E.; Ghozali M.; Kartika W.; Melati R.; Yulianti S.; et al. Molecular Weight Distribution of Lactic Acid Oligomer from the Polycondensation Without Catalyst and Its Application for the Starch Modification. J. Polym. Environ. 2024, 32 (4), 1892–1906. 10.1007/s10924-023-03092-6.

[ref19] Rahmayetty; Sukirno; Gozan M. Effect of Polycondensation Temperature to Oligomer Yield and Depolimerisation Side Reaction. World Chem. Eng. J. 2018, 2 (1), 12–16.

[ref20] Wardhono E. Y.; Pinem M. P.; Kustiningsih I.; Effendy M.; Clausse D.; Saleh K.; Guénin E. Heterogeneous Deacetylation Reaction of Chitin under Low-Frequency Ultrasonic Irradiation. Carbohydr. Polym. 2021, 267 (March), 118180 10.1016/j.carbpol.2021.118180. 34119148

[ref21] Yee D.; Li Z.; Yi D.; Low S.; Ying S.; Manickam S.; Wei K.; Hong Z. Ultrasonics Sonochemistry Ultrasonic Cavitation : An Effective Cleaner and Greener Intensification Technology in the Extraction and Surface Modification of Nanocellulose. Ultrason. Sonochem. 2022, 90 (June), 106176 10.1016/j.ultsonch.2022.106176. 36174272 PMC9519792

[ref22] Lippert T.; Bandelin J.; Schlederer F.; Drewes J. E.; Koch K. Ultrasonics - Sonochemistry Impact of Ultrasound-Induced Cavitation on the Fluid Dynamics of Water and Sewage Sludge in Ultrasonic Flatbed Reactors. Ultrason. Sonochem. 2019, 55 (January), 217–222. 10.1016/j.ultsonch.2019.01.024. 30712849

[ref23] Achmad F.; Yamane K.; Quan S.; Kokugan T. Synthesis of Polylactic Acid by Direct Polycondensation under Vacuum without Catalysts, Solvents and Initiators. Chem. Eng. J. 2009, 151, 342–350. 10.1016/j.cej.2009.04.014.

[ref24] Rahmayetty; Sukirno; Prasetya B.; Gozan M. Effect of Temperature and Concentration of SnCl2 on Depolymerization Process of L-Lactide Synthesis from L-Lactic Acid via Short Polycondensation. Int. J. Appl. Eng. Res. 2015, 10 (21), 41942–41946.

[ref25] Yigit Y.; Kilislioglu A.; Karakus S.; Baydogan N. Determination of the Intrinsic Viscosity and Molecular Weight of Poly(Methyl Methacrylate) Blends. J. Invest. Eng. Technol. 2019, 2 (2), 34–39.

[ref26] Ulya M.; Agustini R. Pengaruh Suhu Polimerisasi L-Asam Laktat Melalui Metode Ring Opening Polymerization (ROP) Terhadap Karakteristik Polylactic Acid (PLA) Polymerization Temperature Effect Of L-Lactic Acid By Ring Opening Polimerization (ROP) Method On Polylactic Acid. UNESA J. Chem. 2012, 1 (1), 68–74.

[ref27] Alwan A. F. Preparation and Characterization of Polylactic Acid by Ring Opening Polymerization Using Unconventional Heating System. Al-Mustansiriyah J. Sci. 2022, 33 (2), 24–30. 10.23851/mjs.v33i2.1119.

[ref28] Rahmayetty; Whulanza Y.; Sukirno; Rahman S. F.; Suyono E. A.; Yohda M.; Gozan M. Use of Candida Rugosa Lipase as a Biocatalyst for L-Lactide Ring-Opening Polymerization and Polylactic Acid Production. Biocatal. Agric. Biotechnol. 2018, 16 (April), 683–691. 10.1016/j.bcab.2018.09.015.

[ref29] Khouri N. G.; Bahú J. O.; Carvalho B. L.; Wolf R.; Oswaldo V.; Concha C.; Maciel R. Lactic Acid Pre-Treatment for Lactide Production – A Techno- Economic Feasibility Study for the Dehydration Step. Chem. Eng. Trans. 2022, 92 (March), 601–606. 10.3303/CET2292101.

[ref30] Mckenzie T. G.; Karimi F.; Ashokkumar M.; Qiao G. G. Ultrasound and Sonochemistry for Radical Polymerization: Sound Synthesis. Chem. - Eur. J. 2019, 25 (21), 5372–5388. 10.1002/chem.201803771. 30707473

[ref31] Han Y.; Yu X.; Marfavi Z.; Chen Y.; Zhang L.; Chu J.; Sun K.; Li M.; Tao K. A Perspective on Ultrasound-Triggered Production of Reactive Oxygen Species by Inorganic Nano/Microparticles. Adv. NanoBiomed Res. 2024, 4 (9), 2400060 10.1002/anbr.202400060.

[ref32] Kumari B.; Tiwari B. K.; Hossain M. B.; Brunton N. P.; Rai D. K. Recent Advances on Application of Ultrasound and Pulsed Electric Field Technologies in the Extraction of Bioactives from Agro-Industrial By-Products. Food Bioprocess Technol. 2018, 11, 223–241. 10.1007/s11947-017-1961-9.

[ref33] Carpenter J.; Badve M.; Rajoriya S.; George S.; Saharan V. K.; Pandhit A. B. Hydrodynamic Cavitation: An Emerging Technology for the Intensification of Various Chemical and Physical Processes in a Chemical Process Industry. Rev. Chem. Eng. 2016, 33 (5), 433 468 10.1515/revce-2016-0032.

[ref34] Nagatomo D.; Horie T.; Hongo C.; Ohmura N. Ultrasonics Sonochemistry Effect of Ultrasonic Pretreatment on Emulsion Polymerization of Styrene. Ultrason. Sonochem. 2016, 31, 337–341. 10.1016/j.ultsonch.2016.01.010. 26964957

[ref35] Ambrosio-Martín J.; Fabra M. J.; López-Rubio A.; Lagaron J. M. An Effect of Lactic Acid Oligomers on the Barrier Properties of Polylactide. J. Mater. Sci. 2014, 49, 2975–2986. 10.1007/s10853-013-7929-x.

[ref36] Balla E.; Daniilidis V.; Karlioti G.; Kalamas T.; Stefanidou M.; Bikiaris N. D.; Vlachopoulos A.; Koumentakou I.; Bikiaris D. N. Poly (Lactic Acid): A Versatile Biobased Polymer for the Future with Multifunctional Properties—From Monomer Synthesis, Polymerization Techniques and Molecular Weight Increase to PLA Applications. Polymers 2021, 13 (11), 1822 10.3390/polym13111822. 34072917 PMC8198026

[ref37] Groot W.; Van Krieken J.; Sliekersl O.; De Vos S. Poly(Lactic Acid): Synthesis, Structures, Properties, Processing, and Applications, 2010.

[ref38] Chafran L. S.; Paiva M. F.; França J. O. C.; Jos M.; Sales A.; Dias S. C. L. Preparation of PLA Blends by Polycondensation of D,L-Lactic Acid Using Supported 12-Tungstophosphoric Acid as a Heterogeneous Catalyst. Heliyon 2019, 5 (July 2018), e01810 10.1016/j.heliyon.2019.e01810. 31193779 PMC6539807

[ref39] Yi W.-J.; Li L.; Hao Z.; Jiang M.; Lu C.; Shen Y.; Chao Z. Synthesis of L-Lactide via Degradation of Various Telechelic Oligomeric Poly(L-Lactic Acid) Intermediates. Ind. Eng. Chem. Res. 2017, 56, 4867–4877. 10.1021/acs.iecr.6b04082.

[ref40] Pérez J. M.; Ruiz C.; Fernandez I. Synthesis of a Biodegradable PLA: NMR Signal Deconvolution and End-Group Analysis. J. Chem. Educ. 2022, 99, 1000–1007. 10.1021/acs.jchemed.1c00824.

[ref41] Chekal B. P.; Torkelson J. M. Relationship between Chain Length and the Concentration Dependence of Polymer and Oligomer Self-Diffusion in Solution. Macromolecules 2002, 35, 8126–8138. 10.1021/ma020859w.

[ref42] Halász K.; Csóka L. Plasticized Biodegradable Poly(Lactic Acid) Based Composites Containing Cellulose in Micro- and Nanosize. J. Eng. 2013, 2013 (1), 329379 10.1155/2013/329379.

[ref43] Ni’mah H.; Rochmadi R.; Woo E. M.; Widiasih D.; Mayangsari S. Preparation and Characterization of Poly(L-Lactic Acid) Films Plasticized with Glycerol and Maleic Anhydride. Int. J. Technol. 2019, 10 (3), 531–540. 10.14716/ijtech.v10i3.2936.

[ref44] Pérez-Fonseca A. A.; Fuentes-Talavera F. J.; et al. Crystallinity and Impact Strength Improvement of Wood-Polylactic Acid Biocomposites Produced by Rotational and Compression Molding. Maderas: Cienc. Tecnol. 2021, 23, 1–16. 10.4067/s0718-221x2021000100436.

[ref45] Maharana T.; Mohanty B.; Negi Y. S. Melt–Solid Polycondensation of Lactic Acid and Its Biodegradability. Prog. Polym. Sci. 2009, 34 (1), 99–124. 10.1016/j.progpolymsci.2008.10.001.

[ref46] Hwang S. W.; Lee S. B.; Lee C. K.; Lee J. Y.; Shim J. K.; Selke S. E. M.; Soto-Valdez H.; Matuana L.; Rubino M.; Auras R. Grafting of Maleic Anhydride on Poly(L-Lactic Acid). Effects on Physical and Mechanical Properties. Polym. Test. 2012, 31 (2), 333–344. 10.1016/j.polymertesting.2011.12.005.

[ref47] Liu R.; Zhao F.; Yu X.; Naito K.; Ding H.; Qu X.; Zhang Q. Synthesis of Biopolymer-Grafted Nanodiamond by Ring-Opening Polymerization. Diamond Relat. Mater. 2014, 50, 26–32. 10.1016/j.diamond.2014.08.011.

